# Lignin-Based Hydrogels for the Delivery of Bioactive Chaga Mushroom Extract

**DOI:** 10.3390/polym16060807

**Published:** 2024-03-14

**Authors:** Aditi Nagardeolekar, Prajakta Dongre, Biljana M. Bujanovic

**Affiliations:** 1 Saint-Gobain Research North America, 9 Goddard Rd, Northborough, MA 01532, USA; aditi.nagardeolekar@saint-gobain.com; 2 Department of Biological Systems Engineering, University of Wisconsin-Madison, Madison, WI 53706, USA; pdongre@wisc.edu; 3 USDA-FS-Forest Products Laboratory, One Gifford Pinchot Drive, Madison, WI 53726, USA; 4 Department of Chemical Engineering, One Forestry Drive, State University of New York-College of Environmental Science and Forestry, Syracuse, NY 13210, USA

**Keywords:** lignin, chaga extracts, lignin-poly(ethylene)glycol diglycidyl ether hydrogel, absorbent

## Abstract

Lignin-poly(ethylene)glycol diglycidyl ether hydrogels were synthesized from lignin fractions readily extracted during the hot-water treatment of angiosperms: hardwoods, sugar maple and energy-crop willow, monocotyledons, grasses, miscanthus and agriculture residues, and wheat straw. These lignins represent a broad range of chemical structures and properties as a comparative analysis of their suitability to produce the hydrogels as a novel carrier of chaga–silver nanoparticles. The formation of hydrogels was assessed via attenuated total reflectance Fourier-transformed infrared spectroscopy. Then, the hydrogels were observed via scanning electron microscopy and evaluated for their free-absorbency capacity and moduli of compression. Furthermore, a hydrogel produced from kraft lignin and two commercial hydrogels was evaluated to benchmark the effectiveness of our hydrogels. Chaga extracts were prepared via the hot-water extraction of chaga mushroom, a method selected for its relatively higher yields and preserved antioxidizing activities. Hydrogels synthesized with lignins of monocotyledons, wheat straw, and miscanthus were found to be suitable carriers for chaga–silver nanoparticles due to their favorable absorption and release behaviors.

## 1. Introduction

### 1.1. Hydrogels as Delivery Agents for Bioactive Solutions

Hydrogels (HGs) are three-dimensional polymeric networks that are chemically or physically cross-linked with each other and have the intrinsic ability to swell in aqueous solvents, increasing in weight from 10–20% to up to thousands of times their dry weight [1]. The balance between the hydrophilicity of the polymers and the extensive cross-linking between polymers allows them to interact with aqueous solutions or biological fluids while preventing them from dissolving and losing their form. HGs are versatile, relatively modern materials that are amenable to a variety of applications in fields, such as hygiene, agricultural, waste management, coal dewatering, sealing, food additives, pharmaceutical drug delivery, biomedical, biosensor, wound dressing, tissue engineering and regenerative medicine, and diagnostic fields [2]. HGs have traditionally been made from synthetic monomers/polymers, e.g., acrylic acid, acrylamide, N-isopropyl acrylamide, 2-hydroxyethyl methacrylate, vinyl alcohol, and ethylene glycol [3,4,5,6,7,8,9]. Recently, biopolymers have also been explored for HG synthesis, either by themselves or in combination with synthetic components. The most common biopolymers used in HG synthesis are polysaccharides, such as cellulose, xylans, alginates, starch, chitosan, and gelatin, due to their high hydrophilicity [10,11,12,13,14,15]. Lignin can also serve as a renewable and environmentally friendly polymer component for HGs, typically in combination with other synthetic or natural polymers [16]. As opposed to the petroleum-based polymers, apart from being cost-efficient, lignin is biodegradable and CO_2_ neutral and shows little to no cytotoxicity [17]. Due to the variety of functional groups present on its structure, it has the flexibility to be integrated with several copolymers. It is not prone to undergoing explosive chemical reactions, and it is safer to store and handle [16]. As compared to polysaccharide-based biopolymers explored via HG synthesis, it is relatively more resistant to biological attacks due to its antioxidant and antimicrobial properties and, hence, can have a longer shelf-life [17,18]. Additionally, the relative hydrophobicity of lignin is expected to confer additional mechanical stability to the HG and durability in aqueous solutions, thereby prolonging its functional life [3]. Therefore, lignin has been combined with different synthetic polymers, as well as biopolymers, to produce HGs that are applicable in a range of fields (Table 1). Biorefinery lignin, such as the lignin isolated via hot-water extraction pretreatment, is one type of lignin that can be explored for this purpose. This biorefinery lignin is isolated from biomass using relatively mild autohydrolytic conditions and expected to contain a relatively preserved native lignin structure and properties, in contrast to kraft lignin, the dominant industrial lignin today, which is considerably modified during harsh kraft pulping conditions [19]. However, biorefinery lignin is lower in purity compared to kraft lignin, which has a relatively higher lignin content [20]. The synthesis of lignin-based HGs is a relatively new research area, with the oldest reports being made only in the last few decades [21]. Since the publication of these early reports, subsequent reports have described the use of different types of lignins in the synthesis of hydrogels applicable in various fields [22,23,24,25,26,27,28,29,30,31,32,33,34,35,36,37,38,39].

HGs are useful devices for the sustained delivery of suitable agents in the agricultural, pharmaceutical, and fragrance industries. The incorporation of a bioactive agent into the polymeric network of HG results in its sustained release from the HG matrix over an extended time [12]. The use of HGs for sustained release offers the advantage of targeting the release site via strategic placement of the HG and achieving greater dose efficiency by avoiding the non-productive release of the active agent. The ability of HGs to imbibe large amounts of aqueous solutions allows for the administration of fewer doses to the target site, without the harmful effects of overdosing.

Considering these uses, lignin-poly(ethylene glycol) diglycidyl ether (lignin-PEGDGE) HGs were developed for the absorption and subsequent release of chaga–silver nanoparticles. PEGDGE was used as a cross-linker to link lignin oligomers together to form a gel network [40,41,42]. The relatively hydrophilic nature of PEGDGE was expected to balance the relatively hydrophobic nature of lignin, resulting in a HG that is able to adequately interact with solutions while maintaining mechanical stability [24,28,39]. 

### 1.2. Bioactivity of Chaga Mushroom Extracts

Chaga mushroom is the common name for the sterile conk of the basidiomycete fungus *Inonotus obliquus* (Family: *Hymenochaetaceae*). It is generally found in boreal forests in extremely cold climates, such as Russia, China, and North America [43,44]. It mainly grows as a wood-destroying parasite on tree trunks rich in the pentacyclic triterpenoid, a betulinic acid that is the extractive component of bark of trees in the *Betulaceae* family, such as white birch (*Betula papyrifera*), yellow birch (*Betula alleghaniensis*), black birch (*Betula nigra*), and ironwood (*Carpinus caroliniana*) [45]. In general, medicinal mushrooms, including chaga, are currently used as dietary supplements and pesticides [46]. Chaga, in particular, has been employed in traditional home remedies for centuries [43]. Antioxidizing, hepatoprotectant, immunomodulatory, and antimicrobial activities are the most common biological activities attributed to chaga, while polysaccharides, alkaloids, phenolic compounds, sterols, and triterpenoids are the most commonly recognized active ingredients in the extracts [46,47,48,49]. Most of the biological activities of these compounds come from their radical quenching ability (antioxidant activity) and their ability to diminish oxidative stress in cells attributed to reactive oxygen species (ROS), such as superoxide anion and hydroxyl radicals, leading to stress-induced diseases, such as cancer, autoimmune diseases and Parkinson’s disease [46]. However, the chemical composition and biological activity vary depending on the extraction method, age of harvested chaga, and part of chaga used [45].

A quick survey of the chaga-based products available at local farmers’ markets and on various websites suggests that a lot of interest has recently been focused on the commercialization of chaga as a dietary health supplement for the management of several chronic diseases. The scientific support for such claims is inconsistent, and there are few reports that investigate the extraction procedures used in the literature [50,51,52,53,54,55,56,57,58,59,60,61,62,63,64]. Current procedures vary in terms of the extraction solvents (aqueous and organic solvents of different polarity), temperature (from room temperature, RT to 100 °C), and duration of extraction (from 30 min to 2 d) used (Table 2). 

### 1.3. Use of Mushroom/Plant Extracts in the Green Synthesis of Nanoparticles

Nanoparticles are a wide class of materials that have at least one dimension that measures less than 100 nm [65]. Among various types of nanomaterials, silver and gold nanoparticles (AgNPs and AuNPs, respectively) have been prominently used in the biomedical field, both for drug delivery in cancer therapy and in biosensing applications [66]. AgNP-based products are an especially popular choice for use in antiseptic sprays and catheters due to the inherent broad-spectrum antimicrobial activity of silver. Apart from the biomedical field, AgNPs are also being explored for use in textiles and food storage containers [67]. For the biological synthesis of AgNPs using plant/mushroom extracts, silver atoms are released in a colloidal form as the result of a redox reaction between reductive components in the extracts (secondary metabolites such as phenols, polysaccharides, alkaloids, terpenoids, and proteins) and silver cations (Ag^+1^) in an aqueous solution [68,69,70]. Chaga extracts can also be employed for this purpose. Besides leading to a reduction of Ag^+1^ ions, the reductive extract components may also remain associated with AgNPs as ‘capping agents’, where they can aid in stabilizing the AgNPs in solution, and used to control their size and shape [69,71,72]. Further, since these compounds often possess bioactive properties themselves, they can have a synergistic effect along with the AgNPs [71]. While a few mushrooms, such as *Agaricus bisporus* [73,74,75,76], *Ganoderma lucidum* [73,75,77], *Pleurotus ostreatus* [75,78], and *Lactarius piperatus* [79], are popular choices for the synthesis of AgNPs, the use of chaga for this purpose has been largely unexplored [80].

Considering the wide variation in the methods employed for the extraction of chaga, as well as the lack of studies on the synthesis of chaga–AgNPs, our primary goal was to develop a protocol for the preparation of chaga extracts for their potential future commercialization. To achieve this aim, different extraction solvents and methods were screened, and the yields, antioxidant capacities, and free phenolic hydroxyl group contents of the resultant extracts were compared. Our second aim was to explore the suitability of selected bioactive chaga extracts for the production of chaga–AgNPs. Thirdly, our aim was to further explore chaga–AgNPs for their suitability for delivery through the lignin–PEGDGE gels, with potential uses as antioxidant and antimicrobial substances in food packaging, agriculture, and wood preservation. 

## 2. Materials and Methods

### 2.1. Chemicals and Materials

Lignins previously recovered from hot-water extracts (RecLs) of angiosperm biomass (Sugar maple RecL, SM; willow RecL, W; miscanthus RecL, MS; and wheat straw RecL, WS) in the pilot plant at SUNY-ESF [20,81,82,83] were explored for the synthesis of HGs. The chemical compositions of the RecLs were determined in our earlier works, and they are presented in Table 3 [20,81,82,83]. Kraft lignin (K) was obtained from Sigma-Aldrich (St. Louis, MO, USA) as a reference lignin, as it was the most abundant technical lignin. PEGDGE (average Mn 500 Da) was purchased from Alfa Aesar (Stoughton, MA, USA), Fisher scientific Inc. (Waltham, MA, USA) and Sigma-Aldrich (St. Louis, MO, USA). Commercial HGs made of acrylamide/potassium acrylate (‘TW’ and ‘CG’) were purchased as commercial controls (Tasty Worms Nutrition Inc., Inverness, FL, USA; eBoot Clear Gel Crystal Beads).

Irregular chunks of 5–6-year-old chaga harvested from birch trees (*Betula alleghaniensis*, Family: *Betulaceae*) in New York state forests were donated by Allegany Trails, Inc. The chunks were ground to #18 mesh particle size, vacuum-dried (~40 °C, ~50.8 kPa) until constant mass was achieved, and used in subsequent experiments.

Other chemicals utilized and their respective vendors are as listed (Table 4). Deionized water (pH ~ 5) was used in all the experiments. All chemicals were used as received without further purification.

### 2.2. Synthesis of the HGs

**Figure 1:**
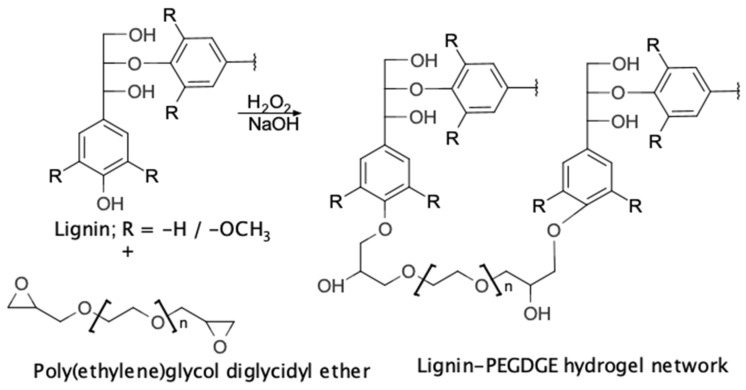
Proposed reaction for synthesis of lignin–PEGDGE hydrogels.

Lignin–PEGDGE HGs were synthesized using the method described in our earlier report [20] (Figure 1). A total of 1 g of RecL (66.7% *w*/*w* of the total weight of polymers in the dry hydrogel) was added to 1.6 mL of 3.3 M sodium hydroxide under constant stirring, followed by the dropwise addition of 0.2 mL of 5% *v*/*v* hydrogen peroxide. The solution was maintained under constant stirring for 24 h. A total of 500 mg of PEGDGE was then added to this lignin solution with constant stirring for another 24 h. The formation of the hydrogel was indicated by the conversion of the liquid solution into a soft solid gel [36]. Four HGs were produced, corresponding to the four separate RecLs mentioned earlier (Viz. SM-PEGDGE HG, W-PEGDGE HG, MS-PEGDGE HG, and WS-PEGDGE HG, respectively). A control HG was produced using kraft lignin (K-PEGDGE HG). 

The weights and dimensions of the HGs were noted using a Mettler—Toledo precision balance tool (AE200, Mettler-Toledo, Columbus, OH, USA) and Vernier calipers, and the approximate density was calculated arithmetically.

### 2.3. Attenuated Total Reflectance Fourier Transform Infrared (ATR-FTIR) Characterization of the Gels

The attenuated total reflectance Fourier transform infrared (ATR-FTIR) spectra of the lignins and HGs were recorded using a PerkinElmer Spectrum Two FTIR spectrometer (PerkinElmer Inc., Waltham, MA, USA) equipped with a Universal Attenuated Total Reflectance Accessory (ATR FTIR spectral analysis). The ATR FTIR spectra were measured in a spectral range from 4000 to 400 cm^−1^ and at a spectral resolution of 4 cm^−1^. For each spectrum, 32 scans were recorded, and the baseline correction and vector-normalization of the spectra were performed. Peak intensities at the peak maxima of the respective bands were determined using PerkinElmer Spectrum software, version 10.5.2 (PerkinElmer Inc., Waltham, MA, USA).

### 2.4. Scanning Electron Microscopy (SEM) Characterization of the HGs [26,86]

A JEOL scanning electron microscope (JEOL JSM IT100LA InTouch Scanning Electron Microscope, JEOL USA Inc., Peabody, MA, USA) was used to observe the morphological characteristics of the HGs.

### 2.5. Measurement of Free-Absorbency Capacity of the HGs

This parameter measured the capacity of the HGs to absorb and retain aqueous solutions, when it is freely swollen, i.e., without any load. It was estimated using two different methods, the modified filtration method and the centrifuge method, in triplicates or more, using gel samples ground to #40 mesh (425 μm). K-PEGDGE HG, TW, and CG were used as the controls. Statistical analysis was performed using RStudio, version 1.2.1335.

- Modified filtration method [87]: A total of 50 mg (W1) of gel was allowed to remain in contact with 50 mL of saline solution for 30 min at RT. The solution was then filtered using a pre-weighed sintered crucible (W0), which was covered to ensure no liquid evaporated during filtration. The crucible was weighed again (W2), and the swelling capacity (Se) was calculated using Equation (1).

Se = (W2 − W0 − W1)/W1(1)

- Centrifuge method [88]: A total of 50 mg (W1) of gel was placed into a tea-bag (made of filter paper, with fabric drawstring, 8 × 10 cm, Tinkee tea-bags, China), which was dipped into 50 mL of saline solution for 30 min at RT. The bag was then removed, and excess solution and inter-particle liquid were removed via centrifugation for 3 min at 250× *g* (Centra-8 Centrifuge, International Equipment Company, Nashville, TN, USA). The combined weight of the bag and the gel (W2) was measured. Same procedure was carried out with an empty bag, and the weight was measured (W0). The swelling capacity of the gel was calculated again using Equation (1).

### 2.6. Determination of the Modulus of Compression

These tests were performed using the Model 100P Universal Resting Machine (Xy software version 4.00.08; TestResources, Shakopee, MN, USA), equipped with 25 N load cell, at a displacement rate of 5 mm/min, log rate of 2/s, and jog rate of 500, at RT. A stress–strain curve was obtained, and the compression modulus was calculated from the slope of the linear portion of the curve. To prepare them for testing, the HGs were cut into disks of 8–10 mm in thickness and 25–30 mm in diameter (as measured using Vernier calipers in at least three places) as soon as they were synthesized. The disks were then lyophilized. The same controls as those described in the earlier sections were used for comparison. These tests were performed in order to measure the mechanical integrity of the HGs during their intended application, and, hence, they were conducted on HG samples soaked in deionized water for 2 h. The tests were continued until the samples showed signs of breaking, indicated by a sharp decrease in the stress. A minimum of three replicate measurements were made. Statistical analysis was performed using RStudio, version 1.2.1335.

### 2.7. Chaga Extraction Solvent Screening

Solvents with different polarities were compared for their extraction efficiency on the extract yield, antioxidant activity (AOA; described below), and PhOH group (described below) content. The relative polarity of the solvents increased as follows: n-Pentane (C_5_H_12_;0.009) < Ethyl acetate (C_4_H_6_O_2_;0.228) < Methanol (CH_3_OH;0.762) < Water (H_2_O;1.00). A common extraction method was used for uniformity and simplicity (ultrasound-assisted bath sonicator extraction; described below). Yields from triplicate extractions were averaged.

### 2.8. Chaga Extraction Method Screening

Common extraction techniques, as described below, were compared for their extraction efficiency. Water was used as a common extraction solvent for uniformity and simplicity.

- Ultrasound-assisted extraction in a bath sonicator (UAE Bath): A total of 2 g of chaga was mixed with 40 mL of solvent in a tightly stoppered Erlenmeyer flask. The flask was suspended in a bath sonicator (40 kHz, Branson 3510 Ultrasonic Cleaner, Branson Ultrasonics Corp, Danbury, CT, USA) completely immersed in the bath maintained at RT. The extraction was continued for a total of 2 h. At midpoint of the extraction period, the solvent was replaced with equal volume of fresh solvent. The two solvent portions were combined, the solvent was evaporated, and the residue was vacuum dried.
- Ultrasound-assisted extraction in a probe sonicator (UAE Probe): A total of 2 g of chaga was mixed with 40 mL of DI water in an Erlenmeyer flask. The sonicator probe was inserted into the flask (Q700, 6Al4V Titanium alloy probe, ¾” diameter, pulse duration of 30 s, duty cycle of 50%; QSonica, Newton, CT, USA). The temperature was maintained under 50 °C by immersing the flask in an ice bath. At the midpoint during the desired extraction period, the solvent was replaced by an equal volume of fresh solvent. The two solvent portions were combined at the end, the solvent was evaporated, and the residue was vacuum-dried.

To compare the effects of extraction intensity (total energy input during UAE) on the extraction yield, AOA, and content of PhOH, two separate extractions were carried out: one for 1 h (total energy input: 152 kJ) and another for 2 h (total energy input: 314 kJ). These extractions were conducted as follows:

- Soxhlet extraction (T204 cm-07): A total of 8 g of chaga was loosely packed in Whatman extraction thimbles placed in a Soxhlet apparatus, attached to an extraction flask (150 mL DI water and boiling chips). The extraction was conducted for 8 h.
- Boiling under reflux: A total of 8 g of chaga was added to a tightly stoppered round-bottom flask containing 150 mL of DI water and boiling chips, fitted with a condenser. The solvent was refluxed for 2 h.
- Hot-water extraction in a Parr reactor (HWE): A total of 4 g of chaga was mixed with 200 mL of water in a Parr reactor (300 mL 4560 Mini bench top reactor, Parr Instrument Company, Moline, IL, USA). To compare the severity of HWE’s impacts on the yield and antioxidant activity, extractions were carried out at various temperatures and durations, expressed collectively as ‘P-factor’ (Equation (2)) [89].

(2) $$P-\mathrm{factor}=\int_{0}^{t}e^{\left(40.48-\frac{15106}{T}\right)}dt$$

where *T* is the extraction temperature (K) and *t* is the extraction time (h). The P-factor has been developed based on the acid-catalyzed cleavage of glycosidic bonds in hardwood xylan (Ea = 125.6 kJ/mol).

### 2.9. Free Phenolic Hydroxyl Group (PhOH) Content

The free phenolic hydroxyl group contents of all samples were determined via the UV ionization difference method [90]. Measurements were conducted in duplicates.

### 2.10. Estimation of Antioxidant Activity (AOA) 

Antioxidant activity was measured by determining the amount of sample needed to quench 50% of the DPPH free radicals present in the solution (DPPH•) [91]. A lower IC_50_ value indicates a higher antioxidant activity. Five observations were made for every sample to compute the IC_50_ value.

### 2.11. Synthesis of the Chaga–AgNPs

Next, 1 mL of chaga HW extract (as extracted at 160 °C, 2 h) was mixed with 19 mL of 0.01 N aqueous solution of silver nitrate [80]. The solution was stirred continuously at RT for 80 min. The development of red/brown color with typical absorption maximum at 439 nm was monitored using a Genesys 10 Series Spectrophotometer. The absorbance was measured every 20 min for up to 80 min. Triplicate observations were made to confirm chaga–AgNP formation.

### 2.12. Absorption and Release of the Chaga–AgNPs Using Lignin–PEGDGE HGs

Approximately 50 mg of lignin–PEGDGE HGs, along with a commercial reference HGs (SM-, W-, MS-, WS-, and K-PEGDGE, along with TW and CG), was allowed to soak into 2 mL of a chaga–AgNP solution (~100 mg/mL) for 1 h. The increase in the weight as a result of the absorption of the chaga–AgNP solution by the HGs was noted. The chaga–AgNP-enriched HGs were then dried in a vacuum oven (~40 °C) for 24 h. They were then resuspended in 6 mL of deionized water with intermittent shaking for 1 h, and the release of chaga–AgNPs from the HG matrix was observed by reading the absorption of the solutions at 439 nm. The percentage amount of chaga–AgNPs released was calculated based on the initial absorbed amount. Duplicate measurements were made, and the average was calculated.

## 3. Results and Discussion

### 3.1. Lignin–PEGDGE HGs

#### 3.1.1. ATR-FTIR Spectral Analysis

**Figure 2:**
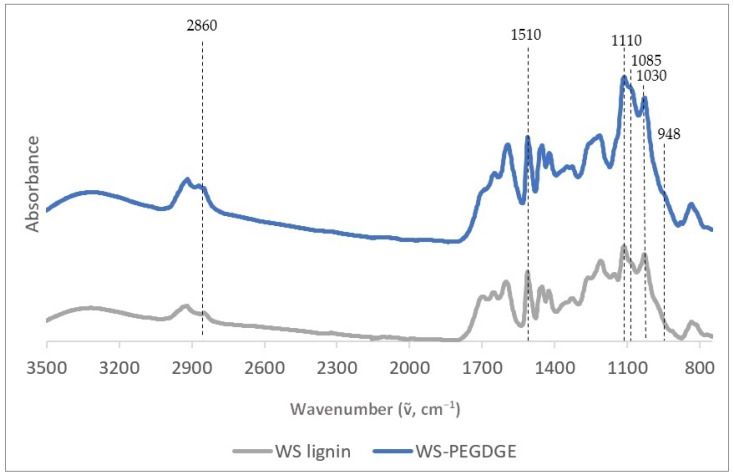
ATR FTIR spectra of wheat straw lignins and hydrogels showing the characteristic bands that indicate the formation of the hydrogel.

**Figure A1:**
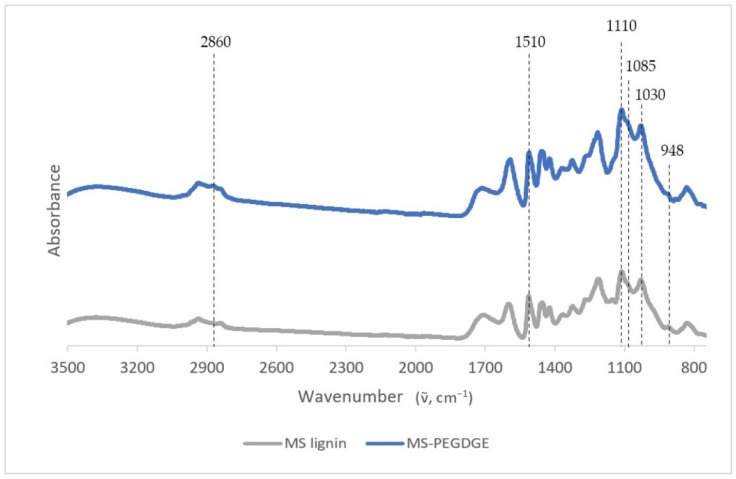
The ATR FTIR spectra of miscanthus lignin and hydrogel showing the characteristic bands that indicate the formation of the hydrogel.

**Figure A2:**
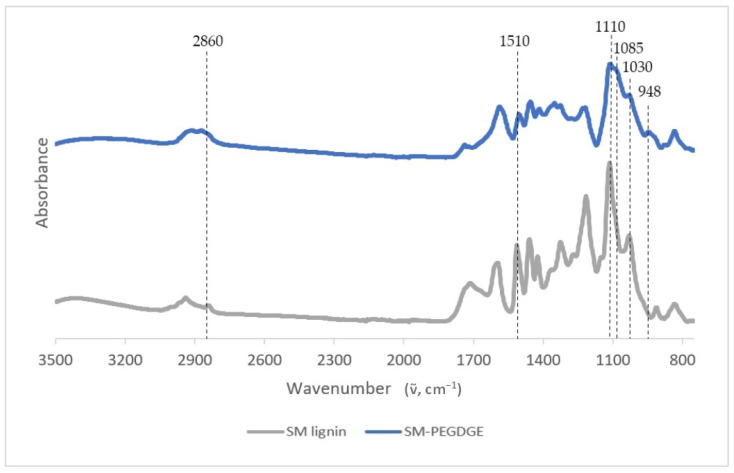
The ATR FTIR spectra of willow lignin and hydrogel showing the characteristic bands that indicate the formation of the hydrogel.

**Figure A3:**
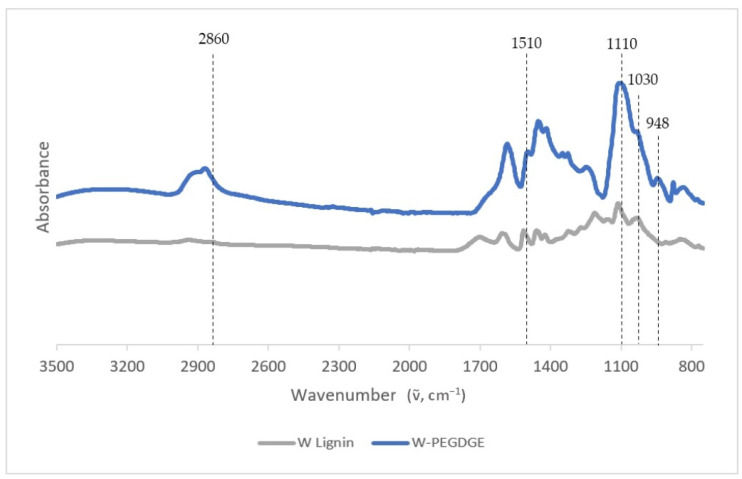
The ATR FTIR spectra of sugar maple lignin and hydrogel showing the characteristic bands that indicate the formation of the hydrogel.

**Figure A4:**
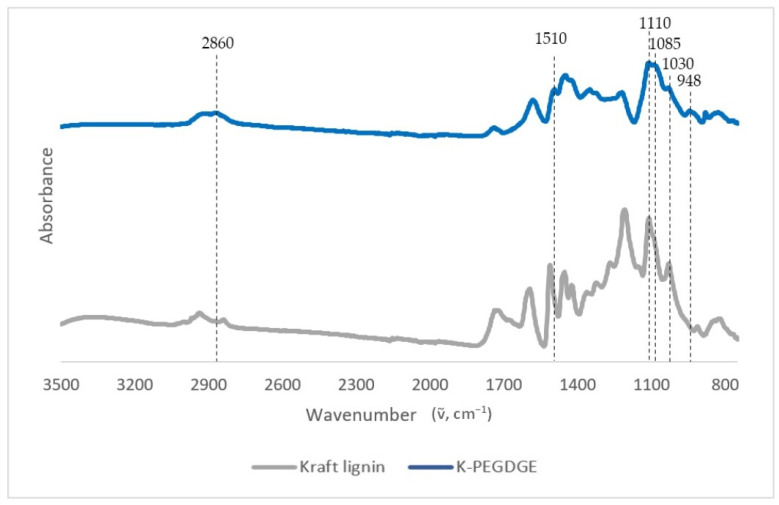
The ATR FTIR spectra of kraft lignin and hydrogel showing the characteristic bands that indicate the formation of the hydrogel.

The ATR FTIR spectra of the formulated HGs show the characteristic bands attributed to the lignin–PEGDGE ethers, confirming their formation (Table 5; [92,93]). Figure 2 shows the ATR FTIR spectra of the WS lignin and hydrogel, whereas the spectra of the MS, W, SM RecLs, and kraft lignins and corresponding HGs are shown in the Appendix A (Figure A1, Figure A2, Figure A3 and Figure A4). Notably, the etherification of all lignins, except W, caused a substantial change in the absorbance at 948 cm^−1^, which corresponds to the C-O-C stretching vibration [92]. However, the extent of modification varies between the lignins, as seen in Table 6, which shows the ratios of the absorbances at characteristic bands relative to that at 1510 cm^−1^ assigned to the aromatic skeletal vibration in lignin [94]. It is interesting to note that WS, despite having a lower content of PhOH than SM, W, and MS, as measured via the UV and periodate methods (Table 3), was able to form more extensive ether linkages with PEGDGE to form the hydrogel network, as indicated by the largest change in the FTIR spectrum (Table 6 and Figure 2). It seems that WS presents an accessible open structure, which, corresponding to a low content in PhOH groups, is abundant in aryl-ether bonds, allowing for the thorough etherification of available PhOH groups. In contrast, kraft lignin, with a relatively higher PhOH content, as measured via the UV and ^31^P-NMR methods (Table 3), showed the least change in its FTIR spectrum after reacting with PEGDGE (Table 6 and Figure 2). This result may be attributed to the widely known condensed nature of kraft lignin, blocking access to PhOH groups as reactive sites [84].

#### 3.1.2. Measurement of Free-Absorbency Capacity

**Figure 3:**
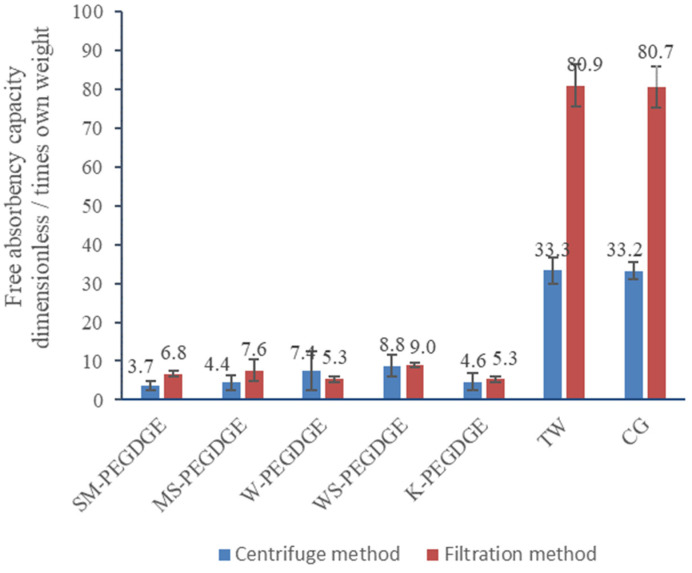
Swelling capacities of HGs, as measured via the centrifuge method and the modified filtration method.

The two methods employed for the measurement of swelling capacity, the modified filtration method and the centrifuge method, produced comparable results for W-, WS-, and K-PEGDGE (*p*-values > 0.05), but not for SM- and MS-PEGDGE HGs, and for the commercial controls, TW and CG (Figure 3). The values obtained from the centrifuge method are generally expected to be lower and more accurate, since this method is more effective at removing loosely bound water from the HG particles [57]. This was true for TW and CG, which showed a swelling capacity measured via the filtration method of more than twice the values measured via the centrifuge method. However, the centrifuge method gave a larger standard deviation between the replicates, possibly due to the variation in the absorption behavior of the tea-bags used in the experiment, unavoidable due to the method constraints. Although both methods showed that the swelling capacities of lignin-containing HGs were inferior to those of the commercial HGs, it should be noted that TW and CG are composed of an entirely synthetic material, much different in composition (acrylamide/potassium acrylate) to our HGs. Between the lignin-containing HGs, K-PEGDGE HG was found to have a swelling capacity equal to that of W-PEGDGE HG and slightly lower than those of SM-, MS-, and WS-PEGDGE HGs, as measured via the modified filtration method. However, these differences were not found to be statistically significant (*p*-value 0.068). WS-PEGDGE was observed to have higher swelling capacity than other lignin-containing HGs, which may be linked to the more efficient formation of its gel network, as seen via FTIR and reported in Section 3.1.1. Overall, the swelling degrees observed for the lignin–PEGDGE HG were in line with the reported values for gels containing lignin in combination with other natural and synthetic polymers [26,36,95,96]. There also have been some reports of lignin-based HGs reaching much higher swelling capacities (ranging from 30 to 389) than the numbers found in our studies [31,96]. These differences likely originate from the types of lignin and the nature of the copolymers used in the synthesis of the HGs, as well as from the various methods used to measure the swelling capacities by different authors. 

#### 3.1.3. SEM Characterization—HG Morphology

**Figure 4:**
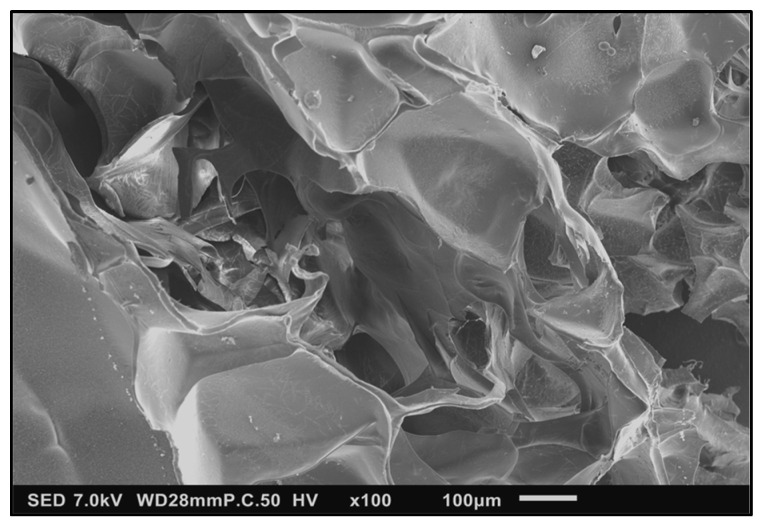
SE micrograph of Au/Pd sputter-coated SM-PEGDGE HG showing a porous, irregular surface. SED, 7 kV, P.C. 50, 100×.

**Figure 5:**
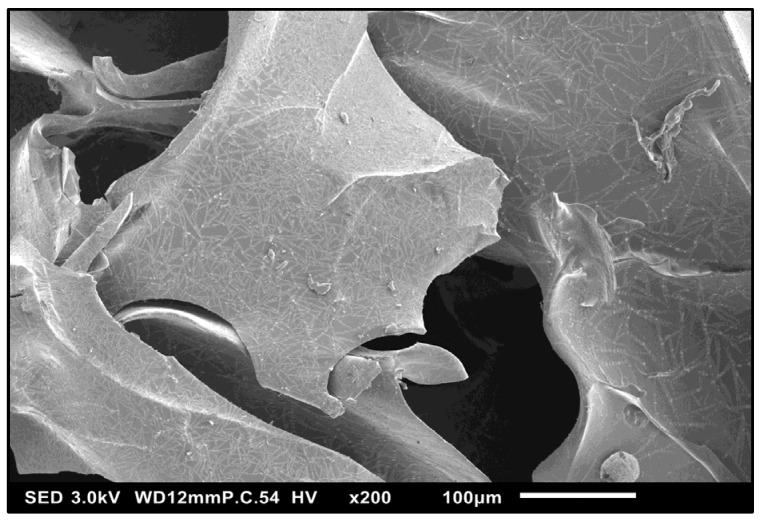
Low-kV micrograph of Au/Pd sputter-coated SM-PEGDGE HG showing surface details. SED, 3 kV; P.C. 54, 200×.

The scanning electron images of lignin–PEGDGE HG showed a porous, irregular surface, with microscopic channels running through the interior (Figure 4). The presence of pores and channels might provide a large surface area for sorption to occur and aid in increasing the permeability and application performance of the gel. Closer inspection of the surface showed the presence of an intricate pattern, which might increase the surface area (Figure 5).

**Figure 6:**
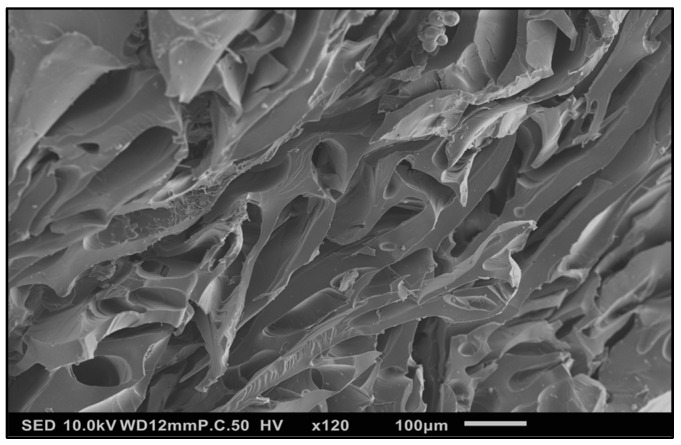
SE micrograph of cryofractured, Au/Pd sputter-coated SM-PEGDGE HG during absorption of ascorbic acid aqueous solution (10 mg/mL). SED, 10 kV; P.C. 50, 120×.

The absorption of an aqueous solution by the HG resulted in the significant distention of the internal structure of the HG (Figure 6), explaining the ability of the gel matrix to swell to accommodate the additional volume.

#### 3.1.4. Determination of Modulus of Compression

**Figure 7:**
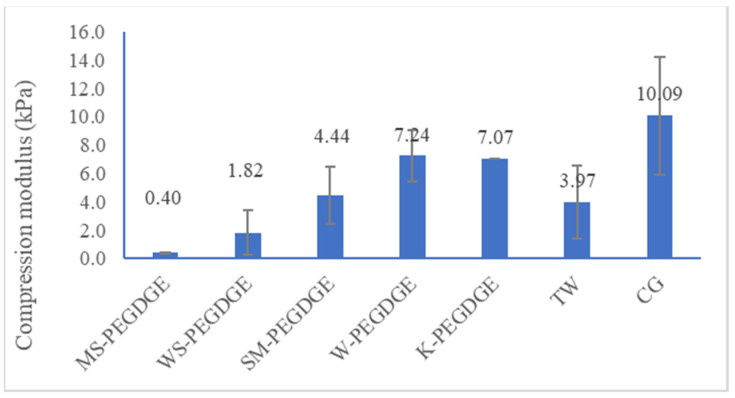
Compression moduli of lignin-PEGDGE HGs and commercial controls, TW, CG.

Among the lignin-containing HGs, MS-PEGDGE HG was found to have the lowest modulus of compression, which was significantly lower than the values observed for SM-, W-, and K-PEGDGE HGs (*p*-value 0.03; Figure 7). Although the commercial reference HGs, TW and CG, exhibited similar swelling capacities (Figure 3), this was not reflected in their moduli of compression, which varied widely (Figure 7). The overall trend observed for swelling capacities was also not reflected in the compression moduli results. A large sampling variability introduced into the calculation based on the nature of the samples might be one of the reasons for these differing results.

### 3.2. Solvent Screening for Extraction of Chaga

#### 3.2.1. Effect of Solvent on Yield

The relatively non-polar solvents (n-pentane and ethyl acetate) were inefficient for the extraction of chaga (yields ≤ 0.01% OD chaga). Between methanol and water, the yield increased with relative polarity (yields 5% and 10% OD chaga, respectively). As expected, polar solvents, such as methanol, ethanol, and water, have been found to be more efficient for the extraction of polar compounds, such as polyphenols (flavonoids), which are thought to be the main contributors to the antioxidant and antitumor effects of chaga, along with some relatively less polar compounds, such as a lanostane triterpenoid and inotodiol [47,63,64]. 

#### 3.2.2. Effects of the Solvent on the AOA and PhOH Contents of Extracts

To further differentiate between the methanolic and aqueous extracts, the AOA and PhOH contents of both the extracts were determined. The AOA values were found to be comparable, with the aqueous extract showing slightly superior AOA values, i.e., IC_50_ 88 μg/mL vs. IC_50_ 106 μg/mL. This result is in line with the reports in which aqueous extracts of chaga were found to possess higher AOA values compared to the alcoholic extracts [63]. However, the aqueous extract showed that it had a more than twice as high PhOH content (0.42 mmol/g) than the methanolic extract (0.19 mmol/g) (Table 7). Taken together, these results suggest that PhOH groups are not the only contributors to the AOA of chaga. Both extracts showed good AOA values, although lower than those of ascorbic acid (vitamin C), which is a natural antioxidant. 

### 3.3. Extraction Method and Screening

#### 3.3.1. Effect of Extraction Method on Yield

HWE (conditions: water-to-biomass ratio (*w/w*) of 50-to-1, 160 °C, 2 h, equilibrium pressure of ~618 kPa) was found to be more efficient for the extraction of chaga out of all techniques (Yields, % OD of chaga: HWE in Parr reactor = 65; UAE (probe, 2 h) = 30; Boiling under reflux = 19; UAE (probe, 1 h) = 14; UAE (bath) = 12; Soxhlet extraction = 10). This result underlines the ability of high temperature and pressure to penetrate the fungal cell walls and give relatively higher yields, as reported in the literature [97,98]. Interestingly, the yield from boiling under reflux (operating at ~100 °C) was slightly lower than that of UAE (probe, 2 h, operating under 50 °C), indicating that temperature was not the only factor that controlled the yield. The presence of high-energy ultrasound waves (total energy input 314 kJ) and replacement of the saturated solvent with the fresh solvent during UAE might have contributed to the higher yield. Additionally, during sonication, although the bulk temperature was controlled by the use of an ice bath, local microregions of intense temperature and pressure were created within the solution due to the cavitation phenomenon [99,100]. This might also have contributed to the higher extraction yields. As expected, the yield obtained from UAE (probe, 2 h) was higher than that obtained from UAE (probe, 1 h) and UAE (bath) due to the relatively lower intensities of the latter two methods.

**Figure 8:**
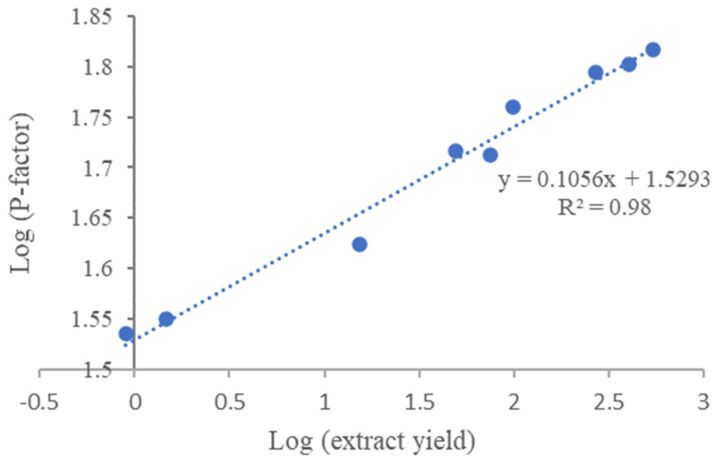
Correlation between P-factor during HWE and chaga extraction yield.

To compare the effects of different severities of HWE on yield, HWE was carried out at different temperatures and times, as expressed by the P-factor (Equation (2)). The log plot of P-factor and chaga extract yield was found to show a good correlation between the two factors (R^2^ 0.98, Figure 8), and it can, thus, serve as a prediction tool for determining the expected yield based on the extraction conditions. 

#### 3.3.2. Effect of the Extraction Method and Intensity on the AOA and PhOH Contents

A closer look was taken at the possible correlation between the extraction severity and the AOA and PhOH contents of the resultant extracts for the top two extraction methods with the highest yields—HWE and UAE (probe).

In the case of UAE (probe), the PhOH contents were found to be almost unaffected (Table 8). For UAE (probe, 2 h), the AOA values decreased (IC_50_ 104 μg/mL) compared to UAE (probe 1 h, IC_50_ 88 μg/mL), indicating the negative effect of more severe conditions on the radical quenching ability of extracts.

In the case of HWE, no correlation was found between the P-factor and AOA and PhOH contents. The IC_50_ ranged between 9.5 μg/mL (P-factor 537) and 177 μg/mL (P-factor 0.9), while the PhOH content ranged between 1 mmol/g (P-factor 99) and 0.53 mmol/g (P-factor 1.5). The corresponding values for each P-factor are available in the Appendix A (Table A1) for more information. These results corroborate previous results suggesting that in addition to PhOH groups, there are other factors that contribute to the AOA values in chaga extracts. 

### 3.4. Synthesis of Chaga–AgNPs

A visual color change from colorless to intense red/brown was observed almost immediately after the addition of silver nitrate solution to the chaga extract prepared via HWE (160 °C, 2 h). The UV absorbance at 439 nm increased for the first 40 min after mixing and then stabilized. The development of red/brown color is a typical feature of AgNPs, attributable to the surface plasmon resonance of the metal nanoparticles [101,102]. This indicates that the antioxidant compounds present in chaga were able to reduce Ag^+1^ ions in the silver nitrate solution to produce chaga–AgNPs.

### 3.5. Absorption and Release of the Chaga–AgNPs Using the Lignin–PEGDGE HGs

**Figure 9:**
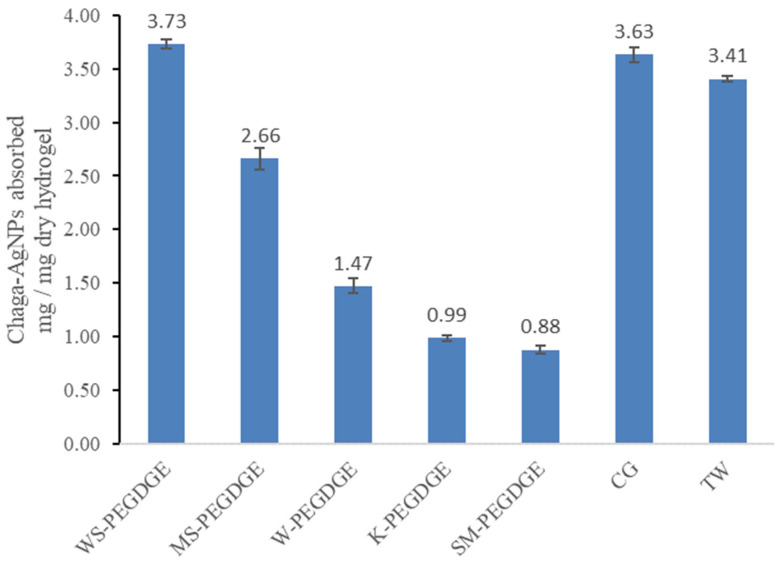
Amount of chaga–AgNPs absorbed by the HGs.

The extent of absorption varied widely between the different HGs (Figure 9). The trend observed for the extent of absorption of chaga–AgNPs by the HGs (WS-PEGDGE > MS-PEGDGE > W-PEGDGE > K-PEGDGE ≈ SM-PEGDGE) showed some similarities and some differences in comparison to the trend seen in the free absorbency capacity measurements for the HGs, as measured via the filtration method (WS-PEGDGE > MS-PEGDGE > SM-PEGDGE ≥ W-PEGDGE = K-PEGDGE) (Figure 3). Consistently higher free absorbency capacities and chaga–AgNPs absorption capacities of WS- and MS-PEGDGE HGs were observed among all lignin-based HGs. Furthermore, WS-PEGDGE was found to be comparable to the commercial HGs TW and CG, as the difference between the average chaga absorption of WS-PEGDGE and the average of the two commercial hydrogels is ~6%, whereas the difference between the average chaga absorption of the commercial hydrogels and all other hydrogels (MS-PEGDGE, W-PEGDGE, K-PEGDGE, SM-PEGDGE) is ~57%. It should be noted that the WS demonstrating a superior performance in the absorption of chaga–AgNPs is characterized by the lowest purity, i.e., the lowest lignin content and the lowest content of PhOH groups among the investigated lignins (Table 3). Again, the higher degree of absorption of WS-PEGDGE may be linked to the more efficient formation of its gel network, as seen via FTIR and reported in Section 3.1.1. More in-depth structural characterization of lignins is required to understand the factors controlling the absorption characteristics of RecL-PEGDGE HGs. The incongruence between the free absorbency capacity and chaga–AgNPs absorption capacity of SM-PEGDGE was noticed. The different protocols used for these two measurements might be the contributing factors for the discrepancy.

**Figure 10:**
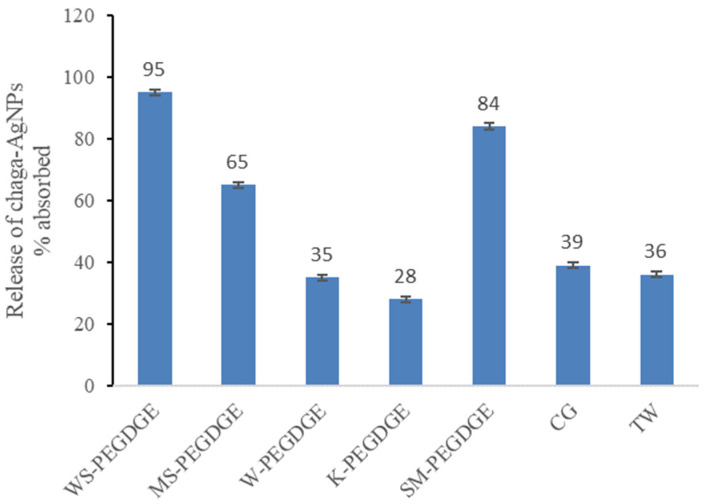
Release of chaga–AgNPs from the HGs.

Similar to the absorption, the release of chaga–AgNPs also varied widely between the HGs (Figure 10). Generally, higher absorption of chaga–AgNPs was found to result in higher release, with the exception of SM-PEGDGE and the commercial HGs. Although SM-PEGDGE had absorbed the lowest amount of chaga–AgNPs (Figure 9), it was found to release a high amount (Figure 10). The opposite was true for the commercial HGs, CG and TW, which released only small amounts of chaga–AgNPs, despite absorbing high amounts. The differential interactions between the HG matrix and chaga–AgNPs might have led to these discrepancies. Thus, WS- and MS-PEGDGE HGs were found to be especially suitable carriers for chaga–AgNPs due to their favorable absorption and release behaviors. K- and W-PEGDGE HGs, TW, and CG were found to be unsuitable carriers due to their unfavorable interactions with chaga–AgNPs, resulting in low release. WS and MS have the lowest lignin contents and the lowest lignin-to-carbohydrate ratios of all lignins (Table 3). Hence, possible factors that may affect the absorption and release rate include the lignin purity; the contents of S-, G-, and H- units; the presence of uncommon residues in lignin (e.g., *p*-coumaroylated and feruloylated end units in MS and WS [103,104,105,106] and *p*-hydroxybenzoate units in W [107]), the PhOH contents of the RecLs; and the extent of ether linkage formation within the hydrogel matrix. Experiments are underway to further study these factors.

## 4. Conclusions

Lignins recovered from hot-water extracts of angiosperms were found to be suitable polymers for the formation of lignin–PEGDGE HGs. Compared to the previous literature on predominantly technical lignin-based HGs and HGs synthesized from lignin isolated under harsher conditions, this study shows the applicability of biorefinery lignin isolated under milder conditions at pilot scale for HG synthesis. HG performance and characteristics probably depend on a combination of the physicochemical and structural features of lignins, such as the chemical composition, polymeric structure, and PhOH content of lignins. Further studies are needed to characterize lignins and their corresponding HGs to understand these specific factors. This flexibility provides opportunities to tailor the HG properties through deliberate lignin selection with a desirable set of features. For the extraction of bioactive compounds from chaga, relatively polar solvents (water and methanol) were found to be good solvents, while hot-water extraction and ultrasound-assisted extraction using a probe sonicator were found to be good extraction methods. Extracts of chaga showed good antioxidant activity. The extraction yield increased with the extraction severity but did not correlate with higher antioxidant activity or free phenolic hydroxyl group content. These results suggested that the antioxidant activities of chaga extracts are probably mediated by a combination of phenolics and non-phenolics. Further, hot-water extracts of chaga (160 °C, 2 h) were found to be capable of reducing Ag^+1^ ions and participate in the green synthesis of chaga–silver nanoparticles. The absorption characteristics of the biorefinery lignin-based HGs for chaga–silver nanoparticles were found to be better than or equal to those synthesized from kraft, the most readily available technical lignin. Although the absorption performance of lignin-based HGs was inferior to that of the commercial HGs, the commercial HGs were limited in functionality.

Overall, these experiments highlight the versatility of lignin as a biopolymer and demonstrate promising results for the commercial utilization of non-woody forest biomass for generating additional revenue.

## Figures and Tables

**Table 1:** Summary of selected reports on lignin-based HGs. Abbreviations: AL: alkali lignin; APS: ammonium persulfate; ATRP: atom transfer radical polymerization; CAS: ceric ammonium sulfate; ECH: epichlorohydrin; KPS: potassium persulfate; LCC: lignin–carbohydrate complex; LCST: lower critical solution temperature; LHW: liquid hot water; LS: lignosulfonates; MCC: microcrystalline cellulose; NMBA: N,N’-methylenebisacrylamide; NIPAM: N-isopropyl acrylamide; OEGDGE: oligo (ethylene glycol) diglycidyl ether; OPGDGE: oligo(propylene)glycol-α,ω-diglycidyl ether; OSL: organosolv lignin; PAM: polyacrylamide; PEGDGE: poly(ethylene glycol) diglycidyl ether; PEGMA: poly(ethylene) glycol methyl ether methacrylate; PVA: polyvinyl alcohol; RICL: radical initiated cross-linking; RISP: radical initiated solution polymerization; SD: swelling degree; SEL: steam explosion lignin; *t*-BHP: *tert*-butyl hydroperoxide.

Lignin Origin	Copolymer/s	HG Synthesis Method	HG Property/Application
AL: Soda [26]	PVA, acrylamide [26]	RISP, KPS initiator [26]	Absorption, SD ~484% [26]
AL: Kraft [26]	PVA, acrylamide [26] NIPAM [27] PEGMA [28]	RISP, KPS initiator [26] ATRP, with lignin macroinitiator created by modifying the PhOH groups [27] Grafting onto lignin by ATRP [28]	Absorption, SD ~147% [26] Thermoresponsive HG, LCST ~32 °C [27] Self-healing smart HG for biomedical applications [28]
AL: LignoBoost [29]	Phenol and formaldehyde [29] Tannin [30]	Conc. alkali [29] Dissolution in alkali in presence of HCHO [30]	Aerogels and cryogels [29] Aerogel for environmental and biomedical applications [30]
AL [31]	Acrylic acid [31]	RICL in alkali, with APS initiator and NMBA cross-linker [31]	Moisture retention in soils [31]
LS [32]	Acrylic acid [32] Polyoxazoline conjugated with triazole [33]	Grafting on LS initiated by laccase/*t*-BHP with NMBA cross-linker [32] Graft polymerization on tosylated lignin in organic solvent [33]	Sorption of cationic dye from wastewater [32] Anti-infective ointment to control chronic inflammation [33]
Acetic acid lignin [34]	NIPAM [34] Isocyanate (-NCO) terminated polyurethane ionomers [35] PEGDGE [24]	Graft copolymerization, initiated by H_2_O_2_, NMBA cross-linker [34] Cross-linking in alkali [24]	Temperature sensitive, LCST ~31 °C [34] Absorption and slow release of (NH_4_)_2_SO_4_ fertilizer [35] Absorption [24]
OSL [36]	PEGDGE [36]	Cross-linking in conc. alkali, initiated by H_2_O_2_ [36]	Absorption SD ~1600% [36]
SEL [37]	MCC [37]	Chemical cross-linking in alkali with ECH and heat [37]	Absorption and controlled release of grape seed polyphenols and vanillin [37]
LHW (200 °C)-extracted lignin [38]	OEGDGE and OPGDGE [38]	Cross-linking in alkali [38]	Aerogel for thermal insulation [38]
LCC [39]	PEGDGE [39]	Dissolution in alkali [39]	Cell carrier for human hepatocytes [39]

**Table 2:** A brief overview of solvents used for the extraction of chaga and corresponding extractive composition and activity. Abbreviations: CH: cyclohexane; EtOH: ethanol; EtOAc: ethyl acetate; HW: hot water; MeOH: methanol; PBS: phosphate buffer saline; RT: room temperature.

Extraction Solvent	Product	Activity	Reference
HW (80 °C, 2 h) or 70% EtOH (70 °C, overnight)	Oxalic acid, gallic acid, protocatechuic, and *p*-hydroxybenzoic acid	Antioxidant, antimicrobial, and anti-quorum sensing	[55]
CHCl_3_ (50 °C, 7 d, percolation)	Spiroinonotsuoxodiol, inonotsudiol, and inonotsuoxodiol	Cytotoxic (antitumor)	[56]
HW (60 °C, 4 h, 3×)	Polysaccharides	Antioxidant	[57]
HW (100 °C, 1 h) MeOH (20%, 50%, 80%, RT, overnight)	Polyphenols	Antioxidant	[58]
CHCl_3_, EtOAc, acetone, EtOH, and H_2_Odist (reflux, 1.5 h)	Phenolics, triterpenoids, polysaccharides	Antioxidant	[47]
Sequential: 95% EtOH (70 °C, 3 h), H_2_Odist. (80 °C, 3 h), precipitated with EtOH	Polysaccharides	Antioxidant	[59]
80–95% EtOH (80 °C-RT, 2–24 h, 2×)	Polysaccharides	Antioxidant	[60,61]
80% MeOH (RT, 2 d, 2×)	Triterpenoids	Cytotoxic (antitumor)	[62]
CH (RT, 90 min, 2×), EtOAc, (RT, 4 d), H_2_O (90 min), H_2_O (100 °C, 15 min)	Betulin, betulinic acid, and inotodiol	Cytotoxic (antitumor)	[63]
PBS (100 °C), 80% and 95% EtOH, and 80% and 95% MeOH (RT)	Polyphenolics	Cytotoxic (antitumor) Antioxidant	[64]

**Table 3:** Chemical compositions of lignins recovered from hot-water extracts of sugar maple, willow, miscanthus, wheat straw [20,81,82,83,84], and softwood kraft lignin [84,85].

Property	Lignin				
	SM	W	MS	WS	K
Total lignin content (% *w*/*w* OD)	86	84.68	81.26	81.03	94.7 *
Acid-insoluble lignin (% *w*/*w* OD)	80.20	80.20	76.30	77.30	89.95 *
Acid-soluble lignin (% *w*/*w* OD)	5.80	4.48	4.96	3.73	4.75 *
Carbohydrates (% *w*/*w* OD)	6.33	2.38	8.68	5.97	1.76 *
Xylan (% *w*/*w* OD)	5.59	1.19	6.36	3.32	0.7 *
Glucan (% *w*/*w* OD)	0.59	1.19	1.16	1.99	0.17 *
Mannan (% *w*/*w* OD)	0.43	0.6	ND	0.48	NA
Arabinan (% *w*/*w* OD)	0.15	ND	1.16	0.66	0.23 *
Ash (% *w*/*w* OD)	0.1	0.2	0.5	1.2	1.69 *
Free phenolic hydroxyl (PhOH) group					
Content (mmol/g lignin)					
UV method	1.43	2.11	1.41	0.99	3.03–4.93
Periodate method	1.95	1.97	1.96	1.19	NA
^31^P-NMR method	NA	NA	NA	2.42	3.01

* Average of values for BioChoice and Indulin AT pine kraft lignin.

**Table 4:** Chemicals used in this study.

Chemical	Vendor
Ammonium persulfate (APS); poly(ethylene) oxide; ilica, fumed; ferulic acid; 2,2-Diphenyl-1-picrylhydrazyl (DPPH)	Sigma-Aldrich
Chloroform	J.T. Baker (Radnor Township, PA, USA)
Dioxane	TCI (Tokyo, Japan)
Hydrogen peroxide; citric acid; silver nitrate solution; trisodium citrate	Fisher Scientific
Methylene violet	MP Biochemicals (Irvine, CA, USA)
*N, N*-Dimethylformamide	VWR, Radnor, PA, USA
Diethyl ether; ethanol; ethyl acetate; methanol; *n*-Pentane	PHARMCO-AAPER, Brookfield, CT, USA
Sodium hydroxide	Macron Fine Chemicals, (Radnor Township, PA, USA)

**Table 5:** Positions and assignments of IR bands characteristic of lignin and lignin hydrogels.

Wavenumber (ṽ) (cm^−1^)	Band Assignment	Refs.
2860	C-H stretching of methyl and methylene groups in PEGDGE	[92]
1510	Aromatic skeletal vibration; G > S	[94]
1110	C-O-C stretching vibration indicating the formation of a PEGDGE lignin ether	[93]
1085	Deformation vibration in secondary alcohols and aliphatic ethers	[92]
1030	C-O-C ether	[92]
948	C-O-C stretching vibration indicating the formation of PEGDGE lignin ether or rocking CH_2_	[92]

**Table 6:** Ratios of absorbances (A) recorded at wavenumbers (ṽ = 2860, 1110, 1085, 1030, and 948 cm^−1^) relative to those at 1510 cm^−1^ for the five RecLs (WS, MS, W, SM, and kraft) and their respective hydrogels (WS-PEGDGE, MS-PEGDGE, W-PEGDGE, SM-PEGDGE, and Kraft–PEGDGE). The numbers in parenthesis indicate the percentage change in the ratio caused by hydrogel formation.

	A_ṽ_/A_1510_				
	ṽ = 2860	ṽ = 1110	ṽ = 1085	ṽ = 1030	ṽ = 948
WS	0.40	1.35	1.13	1.23	0.23
WS-PEGDGE	0.63 (56)	1.91 (42)	2.03 (80)	1.54 (25)	0.77 (238)
MS	0.46	1.49	1.23	1.32	0.48
MS-PEGDGE	0.75 (64)	1.47 (−1)	1.47 (19)	1.24 (−6)	0.74 (53)
W	0.68	1.73	1.36	1.37	0.68
W-PEGDGE	1.73 (155)	2.06 (19)	2.02 (49)	1.33 (−3)	0.57 (−15)
SM	0.34	1.90	1.36	1.11	0.27
SM-PEGDGE	0.68 (98)	1.98 (5)	1.91 (40)	1.38 (24)	0.66 (148)
Kraft	0.64	1.49	1.23	1.02	0.38
Kraft–PEGDGE	0.63 (−2)	1.41 (−5)	1.38 (12)	1.09 (8)	0.67 (75)

**Table 7:** AOA and PhOH contents: chaga extracts and ascorbic acid.

Extraction Solvent	IC_50_ (μg/mL)	PhOH (mmol/g)
Water	88	0.42
Methanol	106	0.19
Ascorbic acid ^1^	33	22 ^2^

^1^ Vitamin C. ^2^ Theoretical value.

**Table 8:** Effect of ultrasound extraction intensity on AOA and PhOH values of aqueous extracts of chaga.

UAE (Probe) Duration (h)	Total Energy Input (kJ)	IC_50_ (μg/mL)	PhOH (mmol/g)
2	314	104	0.49
1	152	88	0.46

## Data Availability

All results obtained in this study are provided in the manuscript.

## Appendix A

**Table A1:** AOA and PhOH contents for hot-water extracts of chaga at different P-factors.

HWE Conditions	P-Factor	IC_50_ (μg/mL)	PhOH (mmol/g)
100 °C, 60 min	0.9	177	0.68
120 °C, 120 min	15.417	25	0.57
140 °C, 90 min	74.36	115.5	0.94
160 °C, 60 min	268.6	18	0.77
100 °C, 90 min	1.47	200	0.53
140 °C, 60 min	49.58	16.6	0.65
140 °C, 120 min	99.17	14	1
160 °C, 120 min	537.17	9.5	0.75
160 °C, 90 min	402.87	15	0.76
